# Characterization and Outcomes of SARS-CoV-2 Infection in Overweight and Obese Patients: A Dynamic Comparison of COVID-19 Pandemic Waves

**DOI:** 10.3390/jcm11102916

**Published:** 2022-05-21

**Authors:** Roxana Manuela Fericean, Cosmin Citu, Diana Manolescu, Ovidiu Rosca, Felix Bratosin, Emanuela Tudorache, Cristian Oancea

**Affiliations:** 1 Department XIII, Discipline of Infectious Diseases, “Victor Babes” University of Medicine and Pharmacy Timisoara, Eftimie Murgu Square 2, 300041 Timisoara, Romania; rox.manuela@yahoo.com; 2 Doctoral School, ‘’Victor Babes’’ University of Medicine and Pharmacy Timisoara, Eftimie Murgu Square 2, 300041 Timisoara, Romania; 3 Department of Obstetrics and Gynecology, “Victor Babes” University of Medicine and Pharmacy Timisoara, 300041 Timisoara, Romania; 4 Department of Radiology, “Victor Babes” University of Medicine and Pharmacy Timisoara, Eftimie Murgu Square 2, 300041 Timisoara, Romania; dmanolescu@umft.ro; 5 Methodological and Infectious Diseases Research Center, Department of Infectious Diseases, “Victor Babes” University of Medicine and Pharmacy Timisoara, Eftimie Murgu Square 2, 300041 Timisoara, Romania; ovidiu.rosca@umft.ro (O.R.); felix.bratosin7@gmail.com (F.B.); 6 Center for Research and Innovation in Precision Medicine of Respiratory Diseases, “Victor Babes” University of Medicine and Pharmacy Timisoara, Eftimie Murgu Square 2, 300041 Timisoara, Romania; tudorache_emanuela@yahoo.com (E.T.); oancea@umft.ro (C.O.)

**Keywords:** COVID-19, SARS-CoV-2 infection, obesity, overweight, disease severity

## Abstract

There are few data on the dynamics of SARS-CoV-2 viral manifestations in obese and overweight persons during each of the five waves that occurred in Romania during the last two years. As such, the purpose of this research was to characterize the variance in case severity, symptomatology, ICU hospitalizations, and mortality among overweight and obese individuals infected with the SARS-CoV-2 virus. We included 250 overweight and obese patients admitted to hospital with COVID-19, where 50 patients were selected from each of the five pandemic waves that existed in Romania until March 2022. A total of 113 patients with normal body mass index were included in the study. They were matched with overweight and obese patients by age, gender, and cardiovascular comorbidities to avoid the effect of confounding factors. Between the five waves of the COVID-19 pandemic in Romania, the present investigation found substantial changes in overweight and obese patient features. Obesity increases the risk of hospitalization, severe complications, and mortality from COVID-19. However, this unique demographic is disproportionately affected by obesity-related comorbidities, which contribute to these adverse outcomes. We advocate for the development of new guiding principles for the formulation of healthcare strategies aimed at high-prevalence special populations such as overweight and obese individuals, while also promoting pandemic containment and avoiding the recurrence of pandemic waves with the same guidelines that proved detrimental in terms of economic and human life loss.

## 1. Introduction

The SARS-CoV-2 virus had a prompt worldwide spread since it was first identified in December 2019 in Wuhan City, Hubei Province, China [1,2,3]. The new coronavirus has a genetic sequence similar to that of the first coronavirus SARSCoV, the virus that caused the 2003 severe acute respiratory syndrome (SARS) pandemic [4,5]. They both include the coronavirus spike (S) protein. The cellular serine protease TMPRSS2 primes the S protein, allowing it to connect to the angiotensin converting enzyme 2 (ACE2) receptor and gain cellular entry [6]. In comparison to SARSCoV, the new SARS-CoV-2 has a greater affinity for ACE2, making it more easily transmissible [7,8]. Despite the association between SARS-CoV-2 and ACE2, there is presently no evidence that ACE inhibitors or angiotensin receptor blockers are associated with infection [9].

The most often seen symptoms of the 2019 coronavirus disease (COVID-19) are mostly unspecific, such as fever, fatigue and dry cough with high prevalence [10,11,12]. Pneumonia, pulmonary embolism, and acute respiratory distress syndrome (ARDS) are all respiratory symptoms and possible severe manifestations of severe COVID-19 [13,14,15]. All age groups are susceptible to infection, and the median age of hospitalized cohorts ranges between 50 and 60 years [16,17]. Initially, it was thought that men are more likely to be infected with the SARS-CoV-2 virus than women of similar age, and they account for a greater incidence among the hospitalized population requiring critical care, which may represent a difference in illness severity between the sexes [18,19]. However, the latest studies showed debatable results [20]. Additionally, these symptoms and manifestations have persisted more or less continuously during the course of the COVID-19 pandemic’s first 24 months; multiple studies indicate that distinct SARS-CoV-2 variants exhibit considerable differences in symptomatology and infection severity [21].

Several comorbidities have been studied in relationship with severe COVID-19, and it was observed than 25% of hospitalized patients and approximately three quarters of ICU patients with SARS-CoV-2 had at least one comorbid condition [22,23]. Comorbidities such as hypertension, cardiovascular disease, and diabetes mellitus were the most common and often mentioned in reports around the world [24,25], and all are known to be connected with obesity, and indeed, obesity is increasingly recognized as both a comorbidity and a risk factor [26,27]. Obesity prevalence rises with age in both men and women, having significant consequences for global health, since excess weight, as measured by an elevated body mass index (BMI), affects a large proportion of the world’s population, with almost 40% being overweight, and more than 10% obese [28]. Western nations have far higher rates of obesity. For example, in the United States, more than 40% of the total population is obese and another 32% are overweight [29,30], whereas in the United Kingdom, almost 30% of adults are obese and more than 30% are overweight [31,32].

Along with viral replication and antiviral treatment, obesity plays a critical role in COVID-19 development. Obesity was shown to be an independent risk factor for death in a major study and meta-analysis, with overweight patients also being at higher risk of having severe COVID-19 [33]. One possibility is that human ACE2 expression is greater in adipose tissue than in lung tissue [34]. Obese patients may have impaired lung function, a poor response to artificial ventilation, and a variety of other problems [35]. The growing body of research has concentrated on obesity and the adverse consequences associated with severe COVID-19.

Comprehensive investigations indicated that up to fifty percent of COVID-19 victims had metabolic and vascular abnormalities, establishing a clear relationship between COVID-19 and the metabolic and endocrine systems. Thus, not only are individuals with metabolic dysfunction at a higher risk of having severe COVID-19, but infection with SARS-CoV-2 may also result in the establishment of diabetes or a worsening of preexisting metabolic diseases [36]. At the molecular level, these effects can be explained by the effects of metabolic syndrome on mitochondria and inflammation. There are several pathways involving mitochondria and their functions in inflammation that may shed light on why SARS-CoV-2 affects overweight and obese patients. Several pathways connect aged mitochondria with decreased immunity, including over-stimulated or persistent inflammatory responses with interferon and cytokine production, mitochondrial biogenesis, and interference with apoptosis and mitophagy [37]. To our knowledge, however, data are few on the dynamics of SARS-CoV-2 viral manifestations in overweight and obese individuals during each of the five waves that were present in Romania in the past two years. Therefore, this study aimed to describe the variation in case severity, symptomatology, ICU admissions and mortality in the population of overweight and obese patients infected with the SARS-CoV-2 virus.

## 2. Materials and Methods

### 2.1. Study Design and Ethics

A multidisciplinary research protocol was designed as a retrospective cohort study of overweight and obese patients with COVID-19 that were admitted to hospital during a period that included five pandemic waves. The study took place in a tertiary hospital, and the setting comprised the departments of infectious disease, pulmonology, and radiology of the Infectious Diseases and Pulmonology Hospital, “Victor Babes” in the period starting March 2020 until March 2022.

The research protocol was approved by the Ethics Committee of the “Victor Babes” University of Medicine and Pharmacy in Timisoara, Romania. The study was conducted according to the guidelines of the Declaration of Helsinki and approved by the Ethics Committee of “Victor Babes” Clinical Hospital for Infectious Diseases and Pulmonology in Timisoara, which operates in accordance with Article 167 of Law No. 95/2006, Art. 28, Chapter VIII of Order 904/2006; with European Union’s GCP Directives 2005/28/EC, the International Conference on Harmonization of Technical Requirements for Registration of Pharmaceuticals for Human Use; and with the Declaration of Helsinki—Recommendations Guiding Medical Practice. It was approved on 28 February 2022, with approval number 05.

### 2.2. Inclusion Criteria and Variables

A database and patient paper record search was conducted to determine the adult overweight patients and those suffering from obesity admitted to hospital with SARS-CoV-2 infection. The overweight status was considered as a body mass index (BMI) between 25 and 29.9 kg/m^2^, while the obese status was regarded as a BMI higher than 29.9 kg/m^2^. The COVID-19 status was defined by a positive polymerase chain reaction test (PCR) from oropharyngeal and nasal swabs. A predefined case report form was used to gather demographic, clinical, and outcome data from electronic medical records. The collected patient data were further stratified by the pandemic wave when the hospital admission was registered. The first wave was considered as the period between March and October 2020 when the main circulating variant was Wuhan-Hu-1 (NCBI Reference Sequence: NC_045512.2) [38]. The second COVID-19 wave was between October 2020 and February 2021, with the Clade variants (S:D614G) as the main circulating variants [39]. The third pandemic wave in Romania was between February and July 2021, having the Alpha (B.1.1.7) variant as the principal circulating viral strain [40]. The fourth wave of the COVID-19 pandemic was between July 2021 and December 2021, when the Delta (B1617.2) variant was causing most of the confirmed infections [41]. Lastly, the fifth pandemic wave in Romania was caused by the Omicron variant between December 2021 and March 2022 [42]. A total of 50 patients were included in the analysis for each wave, totaling 250 overweight and obese patients that were case-matched by age group, gender, and cardiovascular disease after using a stratified random sampling method.

The variables taken into consideration included background data (age, gender, area of residence, occupation, body mass index, smoking status, alcohol use), comorbidities (malignancies, chronic lung disease, cardiovascular disease, cerebrovascular disease, diabetes, autoimmune disease, chronic kidney disease, digestive and liver disease), and COVID-19 treatment. Based on the existing national guidelines [43], clinical picture and documented comorbidities, the COVID-19 patients received antiviral agents, broad-spectrum antibiotics, anticoagulant treatment, steroids and immune modulators for the duration of hospital admission. COVID-19 data (the pandemic wave, clinical severity, imaging severity, oxygen saturation on admission, respiratory rate on admission, heart rate on admission, temperature on admission, duration of hospital stay, duration from symptom onset until hospital admission, viral clearance, intensive care unit admission, duration of ICU stay, oxygen supplementation, mortality), signs and symptoms (cough, fever, dyspnea, headache, digestive symptoms, anosmia/ageusia, fatigue, myalgia/arthralgia, dysphagia), and laboratory data (red blood cells, white blood cells, hemoglobin, hematocrit, platelets, ferritin, erythrocyte sedimentation rate, c-reactive protein, fibrinogen, procalcitonin, D-dimers, interleukin-6, and the international normalized ratio) were assessed.

### 2.3. Statistical Analysis

IBM SPSS v.26 (Armonk, NY, USA: IBM Corp) and MedCalc v.20 (MedCalc Software bv, Ostend, Belgium) were used for statistical analysis. We calculated the absolute (*n*) and relative (%) frequencies of categorical variables and compared their proportions using Chi-square and Fisher’s exact test. The Mann–Whitney test was used to compare non-Gaussian variables that were defined be median and interquartile range (IQR). The mean and standard deviation of continuous variables with a normal distribution were compared using the Student’s t-test (unpaired, independent samples). Finally, a multivariate logistic regression analysis adjusted for confounding variables including age, COVID-19 vaccination, and comorbidities was used to identify independent risk factors for ICU admission and mortality in overweight and obese COVID-19 patients. A significance level of 0.05 was chosen as the alpha value.

## 3. Results

### 3.1. Normal Weight vs. Overweight Patients

A total of 250 overweight patients were included in the study, where 50 of them were infected with SARS-CoV-2 in each of the five waves of the COVID-19 pandemic in Romania. A control group of 113 patients with normal body mass index was included in the analysis, matched by age, gender, and cardiovascular comorbidities. The baseline characteristics comparison of normal weight and overweight patients with COVID-19 admitted to hospital presented in Table 1 did not identify significant differences between proportions of age, gender, area of residence, occupation, and alcohol use disorder in the group of patients with BMI 18.5–24.9 and those with BMI > 24.9. The smoking behavior was more common in overweight patients (51.6% vs. 38.1%, *p*-value = 0.016). Among the studied comorbidities, diabetes mellitus and digestive diseases were found in higher proportions in the overweight patient group (22.4% vs. 12.4%, *p*-value = 0.025), respectively (20.4% vs. 9.7%, *p*-value = 0.012). Regarding the COVID-19 treatment received during hospital admission, there were no important findings when comparing the proportions between overweight and obese patients except for antibiotics that were given with a higher frequency to obese patients (85.6% vs. 77.0%, *p*-value = 0.043).

The comparison of biological parameters and laboratory profiles of the patients included in the current study is presented in Table 2. It was observed that overweight and obese patients were suffering from anemia in higher proportion. The red blood cells and hematocrit were significantly found outside the normal range (65.6% vs. 54.0%, *p*-value = 0.034), respectively (49.2% vs. 27.4%, *p*-value < 0.001). The white blood cell count was also significantly altered compared to that of the group of patients with normal weight (71.6% vs. 60.2%, *p*-value = 0.030).

Table 3 presents the signs and symptoms, as well as COVID-19 outcomes of patients included in the research. It was observed that overweight and obese patients were affected in significantly higher proportion by symptoms related to SARS-CoV-2 infection compared to patients with normal weight, excepting fever, digestive symptoms, anosmia/ageusia, and myalgia/arthralgia. Fatigue was the most common complaint of overweight patients with COVID-19 (86.8% vs. 72.6%, *p*-value < 0.001).

### 3.2. Dynamic Comparison of COVID-19 Pandemic Waves

**Figure 1:**
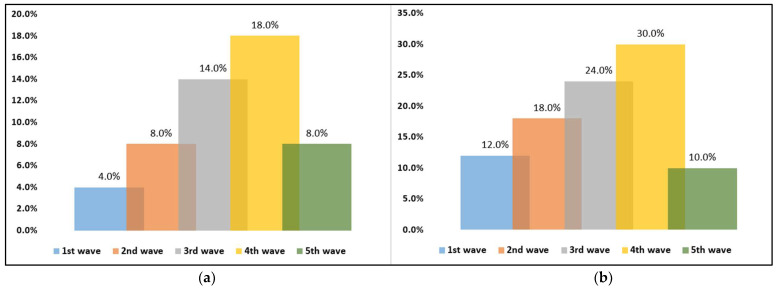
(**a**,**b**) Dynamic comparison of ICU admissions and mortality in overweight and obese patients with COVID-19.

The dynamic comparison of overweight and obese patients with SARS-CoV-2 infection is presented in Table 4. It was observed that during the 4th wave when the Delta variant was the main circulating SARS-CoV-2 strain in Romania, patients were showing statistically significantly higher proportions in ICU admissions and mortality (Figure 1a,b), as well as significantly more altered clinical parameters at hospital admission. The duration of viral clearance was also statistically significantly higher in the 4th wave, with a median of 17 days, compared with 8 days during the first COVID-19 wave (*p*-value < 0.001). It was observed that medication received by the studied patients during the five pandemic waves was not significantly different, excepting the use of antibiotics (*p*-value = 0.007). Additionally, patients started to receive immune modulators from the third wave.

**Table 4:** Baseline characteristics of study participants stratified by COVID-19 pandemic wave.

	1st Wave (*n* = 50)	2nd Wave (*n* = 50)	3rd Wave (*n* = 50)	4th Wave (*n* = 50)	5th Wave (*n* = 50)	*p*-Value *
Severe COVID-19	7 (14.0%)	15 (30.0%)	12 (24.0%)	20 (40.0%)	7 (14.0%)	0.004
Severe imaging features	8 (16.0%)	15 (30.0%)	13 (26.0%)	21 (42.0%)	9 (18.0%)	0.012
Oxygen saturation on admission (<92%)	6 (12.0%)	11 (22.0%)	18 (36.0%)	21 (42.0%)	7 (14.0%)	0.001
Respiratory rate on admission (>20/min)	9 (18.0%)	14 (28.0%)	22 (44.0%)	26 (52.0%)	12 (52.0%)	0.001
Heart rate on admission (>100 bpm)	12 (24.0%)	20 (40.0%)	27 (54.0%)	33 (66.0%)	15 (30.0%)	0.025
Duration of hospital stay	14 (11–18)	9 (7–12)	18 (11–23)	21 (13–25)	9 (7–13)	<0.001
Duration from symptom onset until hospital admission	2 (1–4)	5 (2.2–8.3)	6 (2.3–9.2)	6 (2.1–9.0)	5 (1.9–7.4)	<0.001
Viral clearance	8 (3–13)	12 (4–16)	15 (7–21)	17 (11–23)	9 (4–15)	<0.001
ICU admission	6 (12.0%)	9 (18.0%)	12 (24.0%)	15 (30.0%)	5 (10.0%)	0.002
Duration of ICU stay	7 (3–11)	12 (4–15)	12 (5–16)	14 (9–19)	9 (4–12)	0.009
Severe in-hospital complications	6 (12.0%)	9 (18.0%)	12 (24.0%)	14 (30.0%)	5 (10.0%)	0.021
Oxygen supplementation	22 (44.0%)	27 (54.0%)	37 (74.0%)	45 (90.0%)	17 (34.0%)	<0.001
Mortality	2 (4.0%)	4 (8.0%)	7 (14.0%)	9 (18.0%)	4 (8.0%)	0.001
**COVID-19 treatment**						
Antivirals	50 (100%)	50 (100%)	46 (92.0%)	48 (96.0%)	45 (90.0%)	0.693
Corticosteroids	46 (92.0%)	43 (86.0%)	44 (88.0%)	40 (80.0%)	38 (76.0%)	0.184
Antibiotics	47 (94.0%)	48 (96.0%)	42 (84.0%)	40 (80.0%)	37 (74.0%)	0.007
Anticoagulant	39 (78.0%)	38 (76.0%)	36 (72.0%)	38 (76.0%)	32 (64.0%)	0.528
Immune modulators	0 (0.0%)	0 (0.0%)	28 (56.0%)	29 (58.0%)	26 (52.0%)	0.827

* Data reported as *n* (%) and calculated using Chi-square test and Fisher’s exact unless specified differently.

### 3.3. Risk Analysis

**Figure 2:**
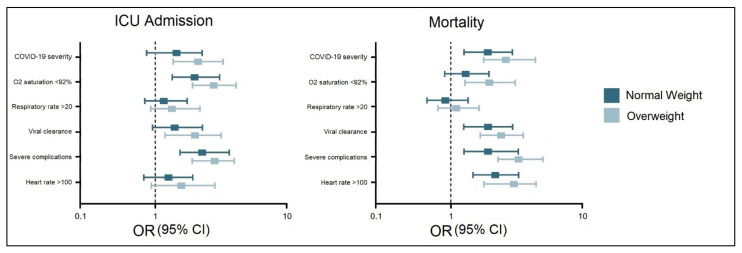
Multivariate risk factor analysis for ICU admission and mortality in normal weight and overweight/obese patients with COVID-19.

The multivariate risk factor analysis for ICU admission and mortality in overweight and obese patients determined that the odds were significantly higher compared to normal weight patients (Figure 2). COVID-19 severity, oxygen saturation below 92% at admission, long viral clearance, and severe complications were significant independent risk factors for ICU admission in overweight patients. In the same manner, COVID-19 severity, long viral clearance, severe complications, and a heart rate higher than 100 beats per minute were identified as independent risk factors for mortality in overweight and obese patients infected with SARS-CoV-2.

## 4. Discussion

The current study determined significant changes in the characteristics of overweight and obese patients that were admitted to hospital between the five waves of the COVID-19 pandemic in Romania. Other studies have attempted to describe the variability of pandemics through viral mutations that occur when millions of individuals are infected. For example, the multi-wave pandemic dynamics were discussed in parallel between the Spanish influenza pandemic of the 20th century and the current and ongoing COVID-19 pandemic [44]. The researchers showed through an epidemic Renormalization Group (eRG) framework that the pace of disease propagation may be viewed as a time-dilation symmetry, and that the last stage of a wave corresponds to achieving a time-invariant state. The short period between two waves is shown to be a symptom of system instability, connected with a near-break of time scale invariance. Their findings indicate that the critical time for containing the next wave of a pandemic is the walking period between waves, during which the number of infections climbs linearly. Thus, preventing or delaying the entrance of the next wave is most effectively accomplished by restricting viral dissemination during this time period [45]. Although accurate methods were employed to forecast the growth of the COVID-19 disease’s case count at the start of the pandemic, it proved challenging to forecast the arrival of a second wave in the autumn of 2020 [46,47].

Other studies analyzed the early public health interventions to control the COVID-19 pandemic waves, although there were no population-specific measurements put in place and described. A South Korean study focused on the second and third pandemic waves and described that the third wave lasted longer than the second and was associated with a greater incidence of case fatalities, which is consistent with our findings [48]. The study concluded that to successfully suppress and manage the COVID-19 pandemic, early and timely interventions with reinforced social distancing regulations should be undertaken. If the first reaction is delayed, the COVID-19 pandemic may grow explosively inside local groups, making future social distancing methods difficult to implement. However, a further development of different SARS-CoV-2 viral strains during the fourth and fifth waves showed that the natural evolution of a pandemic is very hard to control, even though a full-scale vaccination program was successfully implemented in several countries [49,50].

A recent meta-analysis studied the impact of COVID-19 on overweight and obese patients, as well as the risk factors associated with hospitalization, ICU admission, and mortality. The researchers discovered that being overweight increases the risk of COVID-19-related hospitalization but not mortality, but that obesity and severe obesity increase the risk of both hospitalization and death associated with COVID-19 [51]. Additionally, a linear dose–response relationship was seen between obesity categories and COVID-19 results. Moreover, the association’s intensity reduced with time, consistent with the trend of COVID-19’s initial wave. However, there was no given analysis on each separate wave and the dynamics created by the alpha, beta, delta, and more recently, the omicron variant.

The formation of the SARS-CoV-2 alpha, beta, and delta variants of concern was accompanied by succeeding waves of infection, sometimes spanning throughout the whole globe [52]. For instance, the delta COVID-19 strain that was observed in our study to determine more severe infections had greater transmissibility and was accompanied by a larger viral load, a longer duration of infectiousness, and a high incidence of reinfection, owing to its capacity to evade natural immunity [53,54]. As a consequence, the delta strain swiftly became the worldwide prevalent variety. It continued to cause successive waves of infection and remained the major variant of concern in several nations throughout the fourth wave. These concerns regarding decreased vaccine effectiveness due to new variants have altered researchers’ views of the COVID-19 endgame, dispelling the myth that worldwide immunization alone will be sufficient to suppress SARS-CoV-2 infection [55]. The latest SARS-CoV-2 variants have emphasized the critical role of vaccination in conjunction with current public health preventative measures, such as masks, as a route to viral endemicity. Therefore, special populations such as overweight and obese patients who are at higher risk of developing severe complications during COVID-19 should be targeted by nationwide yearly vaccination campaigns such as the influenza vaccination campaign in Romania [56].

An important limitation of the current study is the risk of bias that might be determined by government laws and healthcare guidelines that were constantly changed during the COVID-19 pandemic. Therefore, several differences that were observed between the five waves of the pandemic might be caused by confounding factors such as regulations in COVID-19 patient admission, management, and treatment. Although the impact of obesity on the observed outcomes was justified, these confounding factors can not be ruled out. Moreover, hospitalized COVID-19 patients from the studied population received corticosteroids as the basis of treatment for SARS-CoV-2 infection according to the existing national guidelines at the time. Therefore, this treatment can increase the glycemic index of patients, and more so in overweight and obese that are likely to have metabolic syndrome. It is also worth mentioning the risk of selection bias that might occur in this study, since all patients were admitted at a tertiary hospital. Therefore, it is likely that the presented cases of COVID-19 could be of greater severity than the average. Another drawback of retrospective cohort studies is that since several healthcare providers were engaged in patient care, the assessment of risk variables and outcomes throughout the database is likely to be less precise and consistent than in a prospective cohort study.

## 5. Conclusions

Being overweight or obese determines a higher likelihood of hospitalization, severe complications, or death from COVID-19. However, this special population mostly suffers from obesity-associated comorbidities that contribute towards these negative outcomes. We advocate the creation of new guiding principles for the formulation of healthcare strategies aimed at special populations with high prevalence such as overweight and obese individuals, while also promoting the containment of the pandemic and avoiding the recurrence of pandemic waves with the same guidelines that proved detrimental in terms of economic and human life loss.

## Figures and Tables

**Table 1:** Comparison of baseline characteristics between normal weight and overweight patients with COVID-19.

Variables *	BMI 18.5–24.9 (*n* = 113)	BMI > 24.9 (*n* = 250)	*p*-Value
**Background data**			
Age			0.407
18–40 years	21 (18.6%)	33 (13.2%)	
40–65 years	54 (47.8%)	129 (51.6%)	
>65 years	38 (33.6%)	88 (35.2%)	
Gender (men)	62 (54.9%)	137 (54.8%)	0.990
Area of residence (urban)	68 (60.2%)	143 (57.2%)	0.594
Occupation (employed)	66 (58.4%)	124 (49.6%)	0.119
Smoking	43 (38.1%)	129 (51.6%)	0.016
Alcohol use disorder	9 (8.0%)	17 (6.8%)	0.690
**Comorbidities**			
Malignancy	7 (6.2%)	26 (10.4%)	0.196
Chronic lung disease	11 (9.7%)	39 (15.6%)	0.133
Cardiovascular disease	51 (45.1%)	114 (45.6%)	0.934
Cerebrovascular disease	8 (7.1%)	33 (13.2%)	0.088
Diabetes mellitus	14 (12.4%)	56 (22.4%)	0.025
Autoimmune disease	3 (2.7%)	12 (4.8%)	0.341
Chronic kidney disease	4 (3.5%)	14 (5.6%)	0.402
Digestive and liver disease	11 (9.7%)	51 (20.4%)	0.012
**COVID-19 treatment**			
Antivirals	103 (91.2%)	239 (95.6%)	0.092
Corticosteroids	99 (87.6%)	211 (84.4%)	0.422
Antibiotics	87 (77.0%)	214 (85.6%)	0.043
Anticoagulants	75 (66.4%)	183 (73.2%)	0.184
Immune modulators	28 (24.8%)	83 (33.2%)	0.106

* Data reported as *n* (%) and calculated using Chi-square test and Fisher’s exact unless specified differently; BMI—Body Mass Index.

**Table 2:** Comparison of biological profiles at admission between normal weight and overweight patients with COVID-19.

Variables *	Normal Range	BMI 18.5–24.9 (*n* = 113)	BMI > 24.9 (*n* = 250)	*p*-Value
RBC (millions/mm^3^)	4.35–5.65	61 (54.0%)	164 (65.6%)	0.034
WBC (thousands/mm^3^)	4.5–11.0	68 (60.2%)	179 (71.6%)	0.030
Hemoglobin (g/dL)	13.0–17.0	59 (52.2%)	155 (62.0%)	0.079
Hematocrit (%)	36–48	31 (27.4%)	123 (49.2%)	<0.001
Platelets (thousands/mm^3^)	150–450	38 (33.6%)	104 (41.6%)	0.149
Ferritin (ng/mL)	20–250	33 (29.2%)	96 (38.4%)	0.090
ESR (mm/h)	0–22 mm/hr	75 (66.4%)	177 (70.8%)	0.369
CRP (mg/L)	0–10 mg/L	72 (63.7%)	170 (68.0%)	0.422
Fibrinogen (g/L)	2–4 g/L	69 (61.1%)	171 (68.4%)	0.171
Procalcitonin (ug/L)	0–0.25 ug/L	20 (17.7%)	69 (27.6%)	0.042
D-dimers (ng/mL)	<250	13 (11.5%)	44 (17.6%)	0.139
IL-6 (pg/mL)	0–16 pg/mL	32 (28.3%)	98 (39.2%)	0.045

* Data reported as % outside the normal range, and calculated using Chi-square test and Fisher’s exact unless specified differently; BMI—Body Mass Index.

**Table 3:** Comparison of SARS-CoV-2 infection signs, symptoms, and outcomes between normal weight and overweight patients with COVID-19.

Variables *	BMI 18.5–24.9 (*n* = 113)	BMI > 24.9 (*n* = 250)	*p*-Value
**Signs and Symptoms**			
Cough	72 (63.7%)	188 (75.2%)	0.024
Fever	66 (58.4%)	172 (68.8%)	0.053
Dyspnea	13 (11.5%)	56 (22.4%)	0.014
Headache	9 (8.0%)	42 (16.8%)	0.024
Digestive symptoms	18 (15.9%)	57 (22.8%)	0.134
Anosmia/ageusia	34 (30.1%)	81 (32.4%)	0.661
Fatigue	82 (72.6%)	217 (86.8%)	<0.001
Myalgia/arthralgia	36 (31.9%)	95 (38.0%)	0.259
Dysphagia	7 (6.2%)	49 (19.6%)	0.001
**COVID-19 Outcomes**			
Severe COVID-19	13 (11.5%)	61 (24.4%)	0.004
Severe imaging features	19 (16.8%)	66 (26.4%)	0.045
Oxygen saturation on admission (<92%)	14 (12.4%)	63 (25.2%)	0.005
Respiratory rate on admission (>20/min)	27 (23.9%)	83 (33.2%)	0.074
Heart rate on admission (>100 bpm)	35 (31.0%)	107 (42.8%)	0.032
Duration of hospital stay	14 (9–16)	18 (8–25)	<0.001
Duration from symptom onset until hospital admission	5 (2–8)	5 (1–7)	0.319
Viral clearance	12 (7–19)	15 (6–21)	<0.001
ICU admission	11 (9.7%)	47 (18.8%)	0.029
Duration of ICU stay	7 (3–14)	11 (7–19)	<0.001
Severe in-hospital complications	11 (9.7%)	46 (18.4%)	0.035
Oxygen supplementation	42 (37.2%)	148 (59.2%)	<0.001
Mortality	4 (3.5%)	26 (10.4%)	0.027

* Data reported as *n* (%), and calculated using Chi-square test and Fisher’s exact unless specified differently; BMI—Body Mass Index; ICU—Intensive Care Unit.

## Data Availability

Data available on request.
